# Burnout and Quality of Life Among General Practitioners

**DOI:** 10.1192/j.eurpsy.2026.11681

**Published:** 2026-07-31

**Authors:** A. Kchaou, N. Kotti, F. Dhouib, A. Hrairi, M. Hajjaji, K. J. Hammami

**Affiliations:** Occupational Health, Hedi Chaker University Hospital, Sfax, Tunisia

## Abstract

**Introduction:**

A decline in work-related quality of life among healthcare professionals can lead to reduced empathy and sense of purpose in their job. For primary care physicians, work quality of life is a multidimensional construct influenced by several determinants.

**Objectives:**

This study sought to evaluate the work-related quality of life of general practitioners in the public sector by measuring levels of burnout, compassion fatigue (CF), and compassion satisfaction (CS), as well as to analyze the impact of socio-professional characteristics, CF, and CS on burnout.

**Methods:**

A descriptive cross-sectional study was conducted among public-sector general practitioners in the governorate of Sfax, Tunisia, using an online questionnaire. Collected data included socio-professional information along with responses to the “Professional Quality of Life Scale, version 5” (ProQOL-5).

**Results:**

Seventy-one physicians completed the questionnaire. Moderate levels of burnout, CF, and CS were reported in 81.7%, 64.8%, and 69% of participants, respectively. In the multivariable model adjusted for age, gender, and marital status (adjusted R² = 0.59; p = 0.000), significant independent predictors of burnout were the number of patients consulted daily (β = 0.47, p = 0.000), compassion fatigue (β = 0.45, p = 0.000), and compassion satisfaction (β = -0.70, p = 0.000).

**Conclusions:**

Findings highlight the importance of implementing positive psychology-based strategies for general practitioners in the Sfax public sector, aiming to mitigate burnout and compassion fatigue while enhancing professional well-being.

**Disclosure of Interest:**

None Declared

